# Experimental infection and viral pathogenesis of a human isolate of H5N1 highly pathogenic avian influenza strain in Jersey cows

**DOI:** 10.21203/rs.3.rs-6915991/v1

**Published:** 2025-06-25

**Authors:** Flavio Cargnin Faccin, L. Claire Gay, Dikshya Regmi, Sasha Compton, Teresa Mejías, Juliana Calil Brondani, Lok Joshi, Elizabeth Howerth, Daniela Rajao, Roberto A. Palomares, Daniel R. Perez

**Affiliations:** University of Georgia

## Abstract

Highly pathogenic avian influenza H5N1 viruses have circulated globally since 1996. In recent years, clade 2.3.4.4b H5N1 viruses have crossed the species barrier into multiple species, including dairy cattle, causing a significant decrease in milk production and rapid spread across multiple U.S. states in 2024. Previous studies have demonstrated that Holstein cows are susceptible to H5N1 infection in laboratory settings, mirroring field observations. To investigate whether Jersey cows are susceptible to H5N1 infection and their potential as an experimental model, we inoculated three Jersey lactating cows intranasally and intramammarily with a human H5N1 influenza virus. Following inoculation, milk production rapidly decreased, and milk samples exhibited a colostrum-like yellow appearance. Milk production remained low for at least seven days. All three cows experienced high fever peaks within one day of inoculation. California Mastitis Test scores in milk samples from infected quarters were elevated for several days. Infection was confirmed by high levels of viral RNA detected for several days in external and internal swabs of infected teats, and milk samples. Conversely, low levels of viral RNA were detected in the respiratory tract of infected cows. These findings confirm that Jersey cows are susceptible to H5N1 infection and establish them as a valuable experimental model for studying disease pathogenesis and developing effective vaccines.

## INTRODUCTION

In 1996, the FLUAV lineage A/Goose/Guangdong/1996 (H5N1) was first identified in China, linked to several outbreaks that resulted in mortality and neurological dysfunction in domestic waterfowl ^1^. Highly pathogenic avian influenza (HPAI) H5Nx viruses have continuously evolved through reassortment with other influenza viruses, diversifying into multiple genetic clades and jumping into several species ^2,3^. Notably, clade 2.3.4.4b H5N1 viruses have caused several global outbreaks in recent years, demonstrating an increased potential to infect mammalian species, including raccoons, domestic and wild cats, red foxes, bears, minks, otters, skunks, and sea mammals ^4-6^. From 1996 to 2023, several human infections with H5N1 have been reported, resulting in a case fatality rate exceeding 50%, highlighting its dual threat to both animal and human health ^7^.

On March 25, 2024, a case of HPAI H5N1 was reported in a Texas dairy cow caused by a novel B3.13 genotype, a reassortant virus of an ancestral European 2.3.4.4b strain, and a North American wild bird virus ^8^. Clinical signs in infected cows included decreased rumination rate, mild respiratory signs, lethargy, and, most importantly, decreased milk production with abnormal colostrum-like milk appearance ^9^. Epidemiological and genomic data suggest that cow-to-cow transmission and the interstate movement of animals contributed to the widespread dissemination of the virus, exacerbating the outbreak ^9^. As of June 3rd, 2025, 1073 cases have been confirmed in 17 states across the United States ^8^. While cow-to-human transmission has occurred in isolated cases, all 41 individuals infected during the dairy cow outbreak experienced mild symptoms, including conjunctivitis ^10^. The risk to the human population remains low, as sustained human-to-human transmission has not been documented.

Although Holstein cows remain the dominant breed in the U.S. dairy industry, the Jersey cattle population has been steadily growing, particularly in the southwestern United States ^11^. From 2000 to 2020, the Jersey cow population increased from 3.8–7.9% of the U.S. dairy herd ^12^. Based on the current U.S. dairy cow population of 9.4 million, this represents approximately 600,000 lactating Jersey cows. Previous studies have demonstrated that Holstein lactating cows are susceptible to H5N1 infection, exhibiting mild clinical signs such as mastitis and altered milk appearance ^13^. At the time we conducted the experiments reported here, the susceptibility of Jersey lactating cows to H5N1 infection had not been investigated. To address this, we inoculated three lactating Jersey cows intranasally and intramammarily with a recent human H5N1 virus detected in Texas. Following inoculation, milk production declined rapidly, and milk samples exhibited a colostrum-like yellow appearance, consistent with previous findings. All three cows developed high fever peaks within one day of inoculation, with their temperatures returning to the normal range by day 2 post-infection. California Mastitis Test scores in milk samples from infected quarters were elevated for at least 7 days post-infection. Infection was confirmed by high levels of viral RNA detected in external and internal teat swabs, as well as in milk samples. Conversely, low levels of viral RNA were detected in the respiratory tract of infected cows. Our results demonstrate that lactating Jersey cows are also susceptible to H5N1 infection, making them a valuable experimental model for studying H5N1 disease pathogenesis and developing effective vaccines.

## MATERIALS AND METHODS

### Cells and Eggs

Madin-Darby canine kidney (MDCK) and human embryonic kidney 293T cells (HEK293T) were a kind gift from Robert Webster at St Jude Children’s Research Hospital, Memphis, TN, USA. Cells were maintained in Dulbecco’s Modified Eagles Medium (DMEM, Sigma-Aldrich, St Louis, MO, USA) containing 10% fetal bovine serum (FBS, Sigma-Aldrich), 1% antibiotic/antimycotic (AB, Sigma-Aldrich) and 1% L-Glutamine (Sigma-Aldrich). Cells were cultured at 37°C and 5% CO_2_. Specific pathogen-free (SPF) embryonated chicken eggs (ECEs) used for virus propagation were obtained from Charles Rivers (Wilmington, MA, USA).

### Reverse genetics

Reverse genetics plasmids for wild-type A/Texas/37/2024 (H5N1) were obtained from Twist Biosciences (San Francisco, CA, USA). This virus represents the first reported human exposure to H5N1 from a dairy cow in the United States. Reverse genetics to generate the infectious clone was performed using the 8-plasmid system as described elsewhere ^14^. Briefly, 1.5x10^6^ 293 T cells per well were prepared in Opti-MEM (Fisher Scientific, Hampton, NH, USA) in 6-well plates the day before the transfection. On transfection day, 1 μg of each plasmid was mixed with the TransIT-LT1 transfection reagent (Mirus Bio LLC, Madison, WI, USA) in a ratio of 1 μg plasmid DNA/2 μL of transfection reagent in a final volume of 1 mL of Opti-MEM (Fisher Scientific). The mixture was incubated for 45 min and then used to overlay the 293T cells overnight. The next day, the transfection mixture was replaced with fresh Opti-MEM (Fisher Scientific) containing 1% AB (Sigma-Aldrich). At 24 h post-transfection, 1 μg/mL of tosyl sulfonyl phenylalanyl chloromethyl ketone (TPCK) treated trypsin (Worthington Biochemicals, Lakewood, NJ, USA) was supplemented to the cells. At 72 h post-transfection, the cell supernatant was harvested and centrifuged for 10 min at 3000 rpm. Then, the supernatant was inoculated into 10-day-old SPF eggs. Allantoic fluids were harvested at 48 h post-infection (hpi), centrifuged, aliquoted, and stored at − 80°C.

Virus stocks were titrated by tissue culture infectious dose 50 (TCID_50_) and egg infectious dose 50 (EID_50_). Virus titers were established by the Reed and Muench method ^15^. Virus sequences were confirmed by Sanger sequencing (Psomagen, Rockville, MD, USA).

### Pre-challenge procedures

Approximately 4-year-old lactating Jersey cattle (n = 4) were purchased from a Jersey dairy farm in Georgia. The cows were tested for previous and current influenza virus infections. Additionally, the cows tested negative for the main infectious diseases affecting cattle, including brucellosis, tuberculosis, Johne’s Disease, leptospirosis, bovine viral diarrhea virus, subclinical mastitis, and gastrointestinal parasites, before being transported to the University of Georgia. During a 3-week acclimation period at the Riverbend Farm at the University of Georgia, subcutaneous implantable temperature transponders (Bio Medic Data Systems, Seaford, DE, USA) were inserted under the skin of each quarter, approximately 8 cm above the teat base, to monitor the temperature of individual quarters. In addition, a button-sized thermometer device (Thermochron/Mouser Electronics, Mansfield, TX, USA) attached to a progesterone-free controlled internal drug release (CIDR; Zoetis, Parsippany-Troy Hills, NJ, USA) device was inserted into the cranial vagina to monitor body temperature.

### H5N1 challenge in Jersey cattle

Studies were approved by the Institutional Animal Care and Use Committee (IACUC) at the University of Georgia (Animal Use Protocol A2024 05-012-Y1-A0) to be performed under ABSL3 conditions. A mock cow was moved into the ABSL3 one week prior to the challenge and maintained for three days for mock sample collection. Subsequently, the cow was euthanized for mock tissue collection. Two days before the challenge, the remaining three cows were transferred to the ABSL3 for inoculation. Cows were inoculated with 1x10^6^ TCID_50_/ml of A/Texas/37/2024 (H5N1), administered as follows: 4 ml instilled into each nostril using a DART atomization device (Tri-Anim Health, OH, USA), and 2 ml in each of two quarters (front right [FR], and rear left [RL]), using a teat cannula. After intramammary inoculation, the inoculum was moved up with gentle massage while the teat orifice was occluded to avoid squeezing the inoculum out. The remaining two quarters (front left [FL] and rear right [RR]) were not inoculated.

### Clinical evaluation, swabs, and milk sample collection

Following inoculation, animals were monitored twice daily. Clinical signs were assessed and recorded, including body temperature, quarter temperature, respiratory and rumination rates, nasal and ocular discharge, fecal consistency, dehydration, and body condition score. Additionally, milk production was monitored by weighing the milk produced after milking was completed. The lactating cows were milked once daily in the morning using a portable milker (Mitty Supply, Greenville, GA, USA). Prior to milking, two nasal swabs were collected from the right and left nostrils using a 17-cm polyester sterile swab (Puritan, Guilford, ME, USA). Gloves were changed to collect the next set of samples. Subsequently, another swab was taken from the outside of the RR teat. The RR teat was then cleaned with isopropyl alcohol wipes, and an internal sterile metal mini swab (Puritan) was collected. Swab samples were placed in plastic tubes containing Brain-Heart Infusion media (Sigma-Aldrich) for viral RNA (vRNA) detection. Then, milk samples were manually stripped from the RR teat into separate 15 ml tubes (Thomas Scientific, Chadds Ford Township, PA, USA). These milk samples were evaluated for mastitis using the California Mastitis Test (CMT, PBS Animal Health, Circleville, OH, USA). The remaining milk from each quarter was used for vRNA detection. The same procedure was performed for the FL, RL, and FR teats, in the same order described above, to avoid cross-contamination. Then, teats were pre-dipped and post-dipped with iodine (Tractor Supply, Brentwood, TN, USA) before and after milk collection, respectively, following industry standards. Specifically, for pre-dipping, the teats were disinfected with an iodine solution, allowed to sit for 1 min, and then wiped dry. Following milk collection, the teats were post-dipped with a more concentrated iodine solution containing emollients, such as glycerin and lanolin. The solution was left on the teat until the next milk collection.

### Necropsy and tissue collection

On days 5, 6, and 7 post-infection, cows #2, #3, and #4, respectively, were deeply sedated with a high dose of xylazine followed by intravenous administration of a lethal dose of sodium pentobarbital (Patterson, Saint Paul, MN, USA). At the time of necropsy, all tissues underwent macroscopic evaluation. Table 1 describes all tissues collected for RT-qPCR at necropsy. Tissues collected for RT-qPCR were placed in a 2 ml tube containing 1 ml of RNA later (Sigma-Aldrich).

### Histopathology and immunohistochemistry

Tissue samples were fixed in 10% buffered formalin for at least 72 h, routinely processed, and embedded in paraffin. Three four-micron serial sections were created from each tissue. One section was used for hematoxylin and eosin stain (HE), one for influenza immunohistochemistry (IHC), and one for in situ hybridization (ISH). A board-certified pathologist described the lesions. For IHC, an influenza anti-NP polyclonal antibody (Thermo Fisher, Waltham, MA, USA) was used. The staining intensity was used to estimate the level of viral antigens.

### In situ hybridization (ISH)

ISH was performed using RNAscope 2.5 HD Assay to detect the presence of viral RNA in the tissues. Briefly, the formalin-fixed paraffin-embedded (FFPE) tissue sections were baked at 60°C for 1 h and deparaffinized with xylene. Slides were treated with hydrogen peroxide for 10 min. Target retrieval was performed by boiling slides for 20 min at 100°C using a target retrieval solution (ACD, Newark, CA, USA). The tissue sections were then treated with protease for 15 min at 40°C. Next, slides were incubated with ISH probes for 2 h at 40°C. The V-InfluenzaA-H5N8-M2M1-C1 probe (ACD), which targets segment 7 (matrix protein) of H5Nx clade 2.3.4.4b, was used for detecting viral RNA. The probe targeting the bovine PPIB gene (ACD) was used as a positive control, and a probe targeting the bacterial DapB gene was used as a negative control (ACD). ISH signals were amplified using multiple RNAscope signal amplifiers followed by signal detection using red chromogenic substrate. The slides were counterstained with 50% Gill’s hematoxylin I, and coverslips were mounted on glass slides using EcoMount mounting medium (Fisher Scientific). The slides were allowed to dry at room temperature and imaged at 10X and 20X resolution.

### Virus Titration and rt-qPCR

Tissue homogenates were generated using the Tissue Lyzer II (Qiagen, Hilden, Germany). Briefly, each tube contained the tissue itself, RNA later, and a Tungsten carbide 3 mm bead (Qiagen). Samples were weighted and homogenized for 10 min and then centrifuged at 15,000 g for 10 min. Supernatants were collected, aliquoted, and vRNA was extracted. Briefly, vRNA from tissues, swabs, and milk was extracted using the MagMAXTM-96 viral RNA Isolation kit (ThermoFisher, Waltham, MA, USA). The quantification of vRNA was based on the Influenza A matrix gene using the forward primer M + 25 (5’-AGATGAGTCTTCTAACCGAGGTCG-3’) and the reverse primer M-124 (5’-TGCAAAAACATCTTCAAGTCTCTG-3’) along with the qPCR probe M + 64 (5’-FAM-GGCCCCCTCAAAGCCGA-TAMRA-3’) ^16^. Experiments were carried out in a Quantstudio 3 (Applied Biosystem, Foster City, CA, USA) using qScript XLT One-Step RT-qPCR ToughMix Quantabio (ThermoFisher). Standard curves to correlate quantitative PCR crossing-point values with virus titers were made using a 10-fold dilution from a virus stock of known titer. Virus titers after RT-qPCR were expressed as log_10_ TCID_50_ equivalents/ml or log_10_ TCID_50_ equivalent/g of tissue

### raphs/Statistical Analyses

All data analyses and graphs were performed using GraphPad Prism software version 10 (GraphPad Software Inc., San Diego, CA, USA). Ordinary one-way or two-way ANOVA as needed were performed to calculate P values followed by Tukey’s multiple comparison tests. A P value below 0.05 was considered significant.

## RESULTS

### Infected cows exhibited mild clinical signs following inoculation

**Figure 1:**
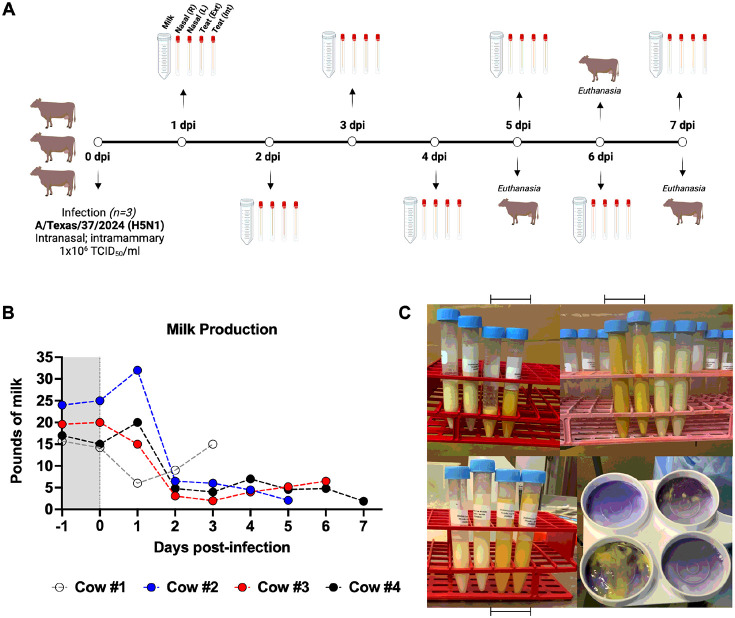
Reduced milk production following H5N1 inoculation. (A) Cows were inoculated intranasally and intramammarily with 1x10^6^ TCID_50_/ml of A/Texas/37/2024 (H5N1). Milk samples, nasal swabs (right and left) and external and internal teat swabs were collected daily. One cow was euthanized for tissue collection on days 5, 6, and 7 post-inoculation (dpi). This figure was created with BioRender.com. (B) Milk production was weighted after each milking session. The figure shows milk production in pounds for Cow #1 (gray, Mock), Cow #2 (blue), Cow #3 (red), and Cow #4 (black). The gray shaded area highlights milk production levels prior to infection. (C) Milk samples from infected quarters are indicated by the bars underneath or above the figures. These samples exhibited a yellow-like appearance and high California Mastitis Test (CMT) scores.

Cows were inoculated intranasally and intramammarily with 1x10^6^ TCID_50_/ml of A/Texas/37/2024 (H5N1) and samples were collected daily (Fig. 1A). Cows were monitored twice daily for clinical signs of infection, including changes in appetite, respiratory and rumination rates, nasal and ocular discharge, fecal consistency, dehydration, and body condition score. No clinical signs were observed in cow #1 (mock) during the experiment. All infected cows developed spontaneous and sporadic cough on days 2 and 3 post-infection and experienced decreased appetite. Notably, cow #3 developed diarrhea on day 1 post-infection, which resolved quickly. Clinical signs resolved rapidly in the following days. We did not observe changes in respiratory or rumination rates, nor did we notice ocular or nasal discharge or dehydration in the infected cows.

### Milk production was drastically reduced upon H5N1 inoculation

Milk production was monitored and weighed daily throughout the experiment. Prior to inoculation, cows produced an average of 20 pounds of milk per day, ranging from 16 to 25 pounds. (Fig. 1B). Although cow #1 (mock) exhibited a drop in milk production upon transport to the BSL-3 laboratory (day 0), production returned to normal levels after 3 days (Fig. 1B). Milk production in infected cows remained relatively unaltered for one day following inoculation, subsequently declining dramatically on day 2, with an average of approximately 5 pounds per cow (Fig. 1B). This low level of milk production persisted throughout the experiment until at least 7 days post-infection for cow #4 (Fig. 1B). As previously described, milk from infected quarters exhibited a yellow, colostrum-like appearance and was associated with elevated California Mastitis Test (CMT) scores (Fig. 1C). Taken together, our findings demonstrate that the H5N1 inoculation in the mammary gland resulted in decreased milk production in all 3 Jersey cows.

### Cows exhibited fever following H5N1 inoculation, along with a temperature decrease in infected quarters

**Figure 2:**
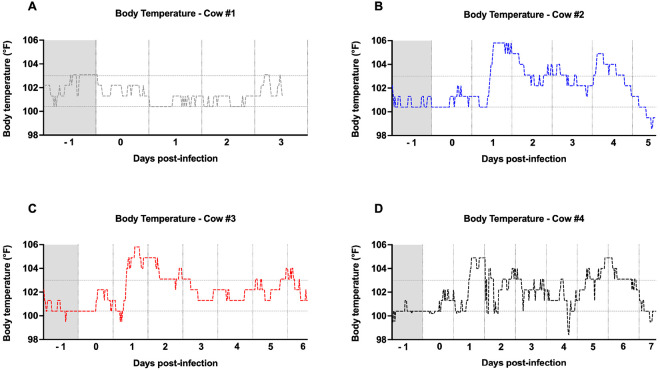
Cows experienced high peaks of fever after H5N1 infection. Body temperature was measured with an i-button intravaginal thermometer and recorded every 30 min throughout the experiment. Each data point represents the temperature measured at a given time by the device. Body temperatures are displayed for cows #1 (A), #2 (B), #3 (C), and #4 (D). The horizontal gray lines represent the normal temperature range for a healthy animal (100.4 °F to 103 °F). The gray shaded area represents the body temperature prior to infection.

Body temperature was monitored using an i-button thermometer attached to a CIDR progesterone-free device. Temperature readings were recorded every 30 min throughout the experiment. Cow #1 (mock) maintained normal temperature levels throughout the experiment (Fig. 2A). Cow #2 exhibited fever episodes one day after inoculation, with temperature peaks reaching 106°F (Fig. 2B). Subsequently, its temperature returned to normal levels with brief fever spikes between days 2 and 3. On day 4, the temperature increased again before returning to baseline prior to euthanasia (Fig. 2C). Consistently, cows #3 and #4 displayed similar patterns, each showing a peak of fever one day post-inoculation (Fig. 2C and D). Temperature peaks indicative of fever was also observed for both cows between days 5 and 6 post-inoculation (Fig. 2C and 2D). In conclusion, these findings indicate that the H5N1 inoculation induced peaks of fever in all three cows.

The temperature of each quarter was measured twice daily using transponders inserted in the udder. All quarters of the mock-infected cow #1 maintained normal temperature levels throughout the experiment, with a slight decrease observed on day 1 **(Fig. S1A).** Interestingly, the temperature of the front right quarter of cow #2 decreased over the study period, while the temperature of the front left quarter remained constant **(Fig. S1B).** Similarly, the temperature of the rear left quarter also decreased over time while the rear right remained constant **(Fig. S1B).** Cow #3 exhibited a general temperature reduction in both front and rear quarters **(Fig. S1C)**. However, a temperature increase observed on day one post-inoculation in the rear quarters coincided with the overall increase in body temperature **(Fig. S1C)**. The temperature in the front right quarter of cow #4 gradually decreased over time, while the temperature in the front left quarter increased as the overall body temperature increased on day one post-inoculation **(Fig. S1D)**. The temperatures in the rear quarters of cow #4 remained relatively stable **(Fig. S1D)**. Overall, while temperatures of infected quarters declined after H5N1 infection, data consistency varied across the three cows.

### Elevated California Mastitis Test (CMT) scores indicate mastitis following H5N1 infection

CMT was conducted on milk samples twice daily. For cow #1 (mock), low CMT scores were observed in all quarters throughout the study **(Fig. S2A).** For cow #2, CMT scores of milk samples from infected teats rapidly increased on day 1 post-infection and remained elevated throughout the study **(Fig. S2B).** Although high CMT scores were also observed in the non-infected rear right teat of cow #2, CMT scores remained normal for the non-infected front left teat **(Fig. S2B).** Similar findings were evident in cow #3, with elevated CMT scores in both infected and non-infected quarters, albeit at lower levels in the latter **(Fig. S2C).** Consistently, high CMT scores were observed in the front right and rear left teats of cow #4, while lower levels were observed in the non-infected quarters **(Fig. S2D).** These data collectively demonstrate that high CMT scores in milk from infected quarters indicate mastitis associated with H5N1 infection.

#### Viral RNA was detected in both external and internal swabs from infected teats of all three cows

**Figure 3:**
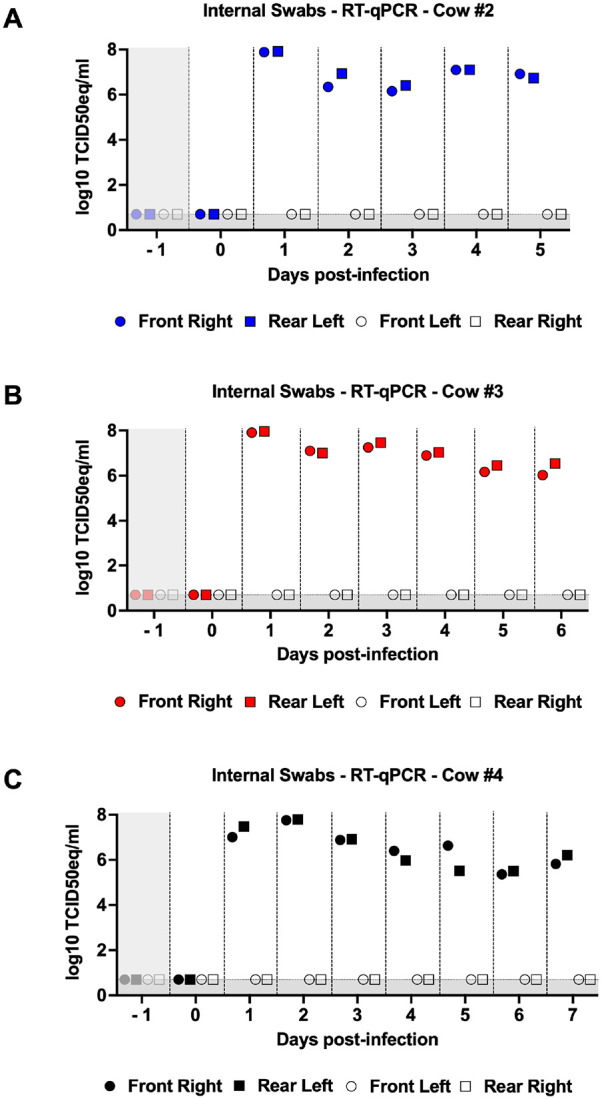
Internal swabs of infected teats contain high levels of viral RNA. Internal swabs of each teat were collected daily. Swabs were titrated by qPCR and viral loads were expressed as log_10_TCID_50eq_/ml. Viral loads for the front right (infected), rear left (infected), front left (non-infected), and rear right (non-infected) quarters of cows #2 (A), #3 (B), and #4 (C). Colored symbols represent infected quarters while clear symbols represent non-infected quarters. Samples with undetected virus titers were assigned the limit of detection value (LOD, 0.699 Log_10_ TCID_50_ equivalent/mL). The gray shaded area represents viral loads prior to infection (day −1).

External and internal swabs were collected daily throughout the study. To avoid redundancy, we did not display the viral load results for cow #1 (mock). Importantly, all samples collected from this cow, including swabs, milk, and tissues, were negative for viral RNA (vRNA). The corresponding Ct values are available in **Supplementary Table 2.** Additionally, this table contains Ct values for all samples collected throughout the study from all cows. Levels of vRNA were observed on the surface of infected teats of cow #2 from day 1 to day 4 post-infection **(Fig. S3A).** Non-infected swab samples remained negative during the study. Similarly, levels of vRNA were observed on the surface of infected teats of cows #3 and #4 **(Fig. S3B and C)**, although no vRNA was detected in external swabs of infected teats from cow #4 on day 4 post-infection **(Fig. S3C).** All three cows exhibited high levels of vRNA in internal swab samples of infected teats throughout the study period, reaching a maximum of 8 log_10_ TCID_50_eq/ml (Fig. 3). While a trend towards decreased viral loads over time was observed in internal swabs for all three cows, all samples remained positive until necropsy days with at least 6 log_10_ TCID_50_eq/ml of vRNA (Fig. 3). Conversely, non-infected internal swab samples remained negative during the study for all animals. These findings strongly support significant viral replication within infected quarters, as demonstrated by the consistently high levels of viral RNA detected throughout the study.

### High levels of viral RNA were detected in milk samples

**Figure 4:**
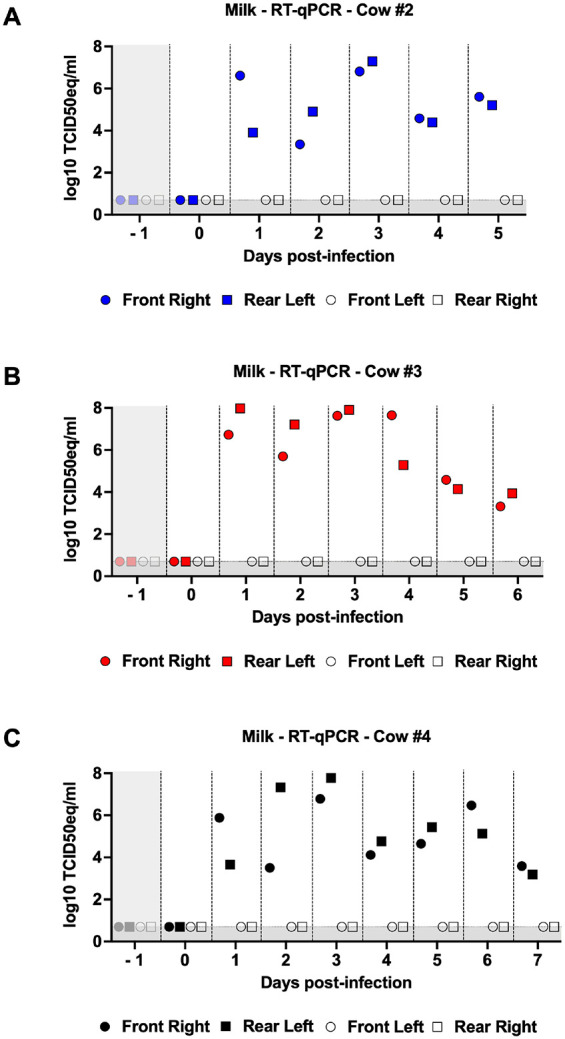
Milk samples contain high levels of viral RNA. Milk samples were collected daily. Samples were titrated by qPCR and viral loads were expressed as log_10_TCID_50eq_/ml. Viral loads for the front right (infected), rear left (infected), front left (non-infected), and rear right (non-infected) quarters of cows #2 (A), #3 (B), and #4 (C). Colored symbols represent infected quarters while clear symbols represent non-infected quarters. Samples with undetected virus titers were assigned the limit of detection value (LOD, 0.699 Log_10_ TCID_50_ equivalent/mL). The gray shaded area represents viral loads prior to infection (day −1).

Consistent with the internal swab data, milk samples from all three cows exhibited high levels of vRNA. Cow #2 displayed a peak vRNA load of 7 log_10_ TCID_50_eq/ml on day 3 post-infection, followed by a decline on days 4 and 5 post-infection (Fig. 4A). vRNA levels in cow #3 rapidly increased 1-day post-infection, reaching peak titers on day 3 post-infection (Fig. 4B). Subsequently, vRNA levels declined in the following days. Consistently, cow #4 showed similar patterns, with the highest vRNA levels observed on day 3 and decreasing thereafter (Fig. 4C). Milk samples from non-infected quarters remained negative throughout the study for all cows. Our data collectively indicate that there is substantial viral replication within milk samples obtained from Jersey cows infected with H5N1.

### Low levels of viral RNA were detected in nasal swabs

Two nasal swabs were collected daily from each cow following infection. Cow #2 exhibited low vRNA levels on days 1 and 2 post-infection, reaching a titer of 4 log_10_ TCID50eq/ml **(Fig. S4A).** Nasal swab samples were negative on subsequent days. Only right nasal swab samples from cow #3 were positive for vRNA for 4 days, with titers decreasing over time **(Fig. S4B).** For cow #4, vRNA was detected exclusively on days 2 and 3 post-infection, remaining negative thereafter **(Fig. S4C).** Collectively, these findings indicate inefficient viral replication within the respiratory tract of Jersey cows infected with H5N1.

#### Viral RNA was detected in mammary gland tissues, with lower levels observed in the respiratory tract

**Figure 5:**
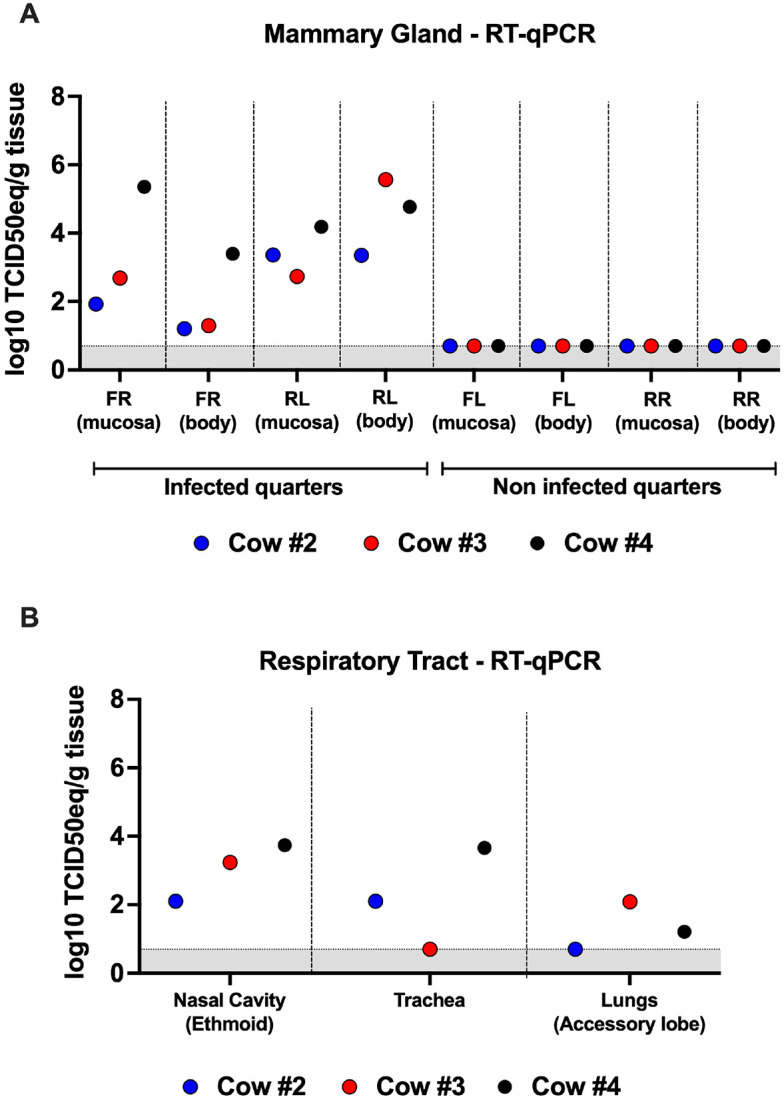
Viral RNA detection in the tissues. One cow was humanely euthanized on days 5, 6, and 7-post-infection for extensive tissue collection. (A) Viral loads in the mammary gland mucosa and body tissues were measured for the front right (FR, infected), rear left (RL, infected), front left (FL, non-infected), and rear right (RR, non-infected) quarters of cows #2 (blue), #3 (red), and #4 (black). (B) Viral RNA distribution in the respiratory tract of infected cows. Samples were titrated by qPCR, and viral loads were expressed as log_10_TCID_50eq_/g tissue. Samples with undetected virus titers were assigned the limit of detection value (LOD, 0.699 Log_10_ TCID_50_ equivalent/g tissue).

On days 5, 6, and 7 post-infection, cows #2, #3, and #4, respectively, were euthanized for extensive tissue collection (Table 1). For the mammary gland tissues, we collected samples from the lactiferous duct (mucosa) and the glandular parenchyma (body). vRNA levels were comparable between these tissues, with cow #4 exhibiting the highest vRNA loads in both for the front right teats (Fig. 5A). Notably, higher vRNA loads were observed in the rear left mammary quarters (Fig. 5.9A). We also collected tissues from the respiratory tract. vRNA was detected in the nasal cavity (ethmoid) of all three cows, and in the trachea of cows #2 and #4 (Fig. 5B). Very low levels of vRNA were detected in the accessory lung of cows #3 and #4 (Fig. 5B). All other tissues tested negative for vRNA. The low levels of vRNA detected in the respiratory tract suggest inefficient viral replication in this tissue. Conversely, the higher vRNA levels found in the mammary gland tissues confirm the tissue tropism of H5N1 for the mammary gland.

#### Histopathological evaluation of mammary gland tissues revealed mastitis and ductitis, concurrent with the detection of elevated protein and viral RNA levels

**Figure 6:**
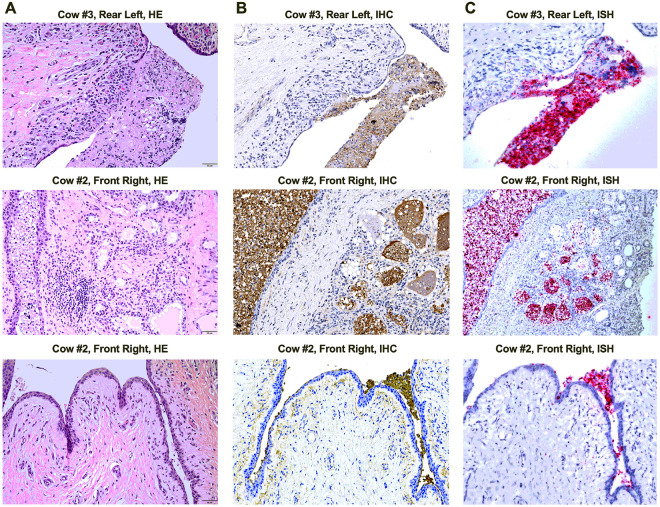
Histopathological evaluation of mammary gland tissues revealed mastitis and ductitis. Tissue samples collected at necropsy were processed for hematoxylin and eosin staining (HE), immunohistochemistry (IHC), and in situ hybridization (ISH). (A) H&E staining of infected tissues from Cows #2 and #3 indicated mastitis and ductitis. (B) IHC staining of infected tissues from Cows #2 and #3 demonstrated viral antigen positive cells. (C) ISH staining of the same tissues showed positive staining for influenza vRNA.

Tissue samples collected at necropsy were processed for HE, IHC, and ISH. The mammary gland tissues exhibited necrotizing mastitis and ductitis. Lobular alterations were diverse, encompassing lobules with alveoli lined by either degenerate or regenerating hyperplastic epithelium, and lobules displaying necrotic alveoli (Fig. 6A and S5A). Necrotic alveoli were denuded of epithelium and filled with necrotic cellular debris, sloughed cells, inflammatory cells (neutrophils and macrophages), and eosinophilic proteinic fluid with lipid vacuoles, indicative of abnormal secretion or milk (Fig. 6A and S5A). The interstitium of affected lobules was infiltrated with low to moderate numbers of lymphocytes, plasma cells, and sometimes neutrophils. Intra- and interlobular ducts were lined by hyperplastic epithelium or, more often, lined by a mild to moderately thick layer of squamous metaplasia (Fig. 6A and S5A). Most ducts were filled with sloughed cells, necrotic debris, and low numbers of inflammatory cells (macrophages and degenerative neutrophils), admixed with abnormal secretion (milk). IHC revealed a variable number of viral antigen positive alveolar cells and, in necrotic areas, viral antigen positive alveolar and ductal luminal contents including sloughed alveolar cells, necrotic debris, and milk (Fig. 6B and S5B). These same areas also showed high viral RNA levels, as confirmed by positive viral RNA ISH staining (Fig. 6C and S5C).

Teat cisternitis was present in all infected mammary quarters, characterized by squamous metaplasia of the epithelial lining and variable-sized foci of erosion or ulceration (Fig. 6A and S5A). Mild infiltrations of lymphocytes, plasma cells, and neutrophils were present in the superficial lamina propria. In infected mammary quarters, IHC staining revealed influenza antigen positivity within scattered to nearly all superficial squamous epithelial cells. These cells were observed sloughing into the lumen, forming viral antigen positive accumulations with necrotic debris (Fig. 6B and S5B). These data confirm the tissue tropism of H5N1 for mammary gland tissue, particularly in the infected quarters of all cows.

## DISCUSSION

H5N1 FLUAVs have historically been known to cause severe disease with high morbidity and mortality among poultry species ^17^. In recent years, H5N1 has consistently crossed the species barrier with unprecedented frequency, expanding its host range. Notably, several mammals not previously considered susceptible to H5N1 infection have been found to be susceptible, raising concerns about potential transmission to humans ^18^. While sporadic detections of antibodies against human H1N1 and H3N2 seasonal influenza A viruses had previously been reported in dairy cows linked to milk production losses ^19^, the cases reported in Texas represent the first documented cases of any FLUAV subtype causing viral mastitis in lactating dairy cows.

Previous studies have investigated the susceptibility of Holstein cows to H5N1 infection, specifically focusing on the role of the mammary gland in transmission and disease development. In one study, inoculation resulted in no nasal shedding but severe acute mammary gland inflammation, high fever, and a drastic decrease in milk production ^20^. Another study reported similar clinical signs in lactating cows, including decreased rumen activity, altered milk appearance, and production losses consistent with field cases of mastitis caused by H5N1 ^13^. High levels of viral RNA in milk, virus isolation, mammary tissue lesions, and seroconversion confirmed the infection ^13^. However, to our knowledge, no existing studies have investigated the susceptibility of dairy cattle breeds to H5N1 infection beyond Holstein cows. As previously mentioned, the Jersey cattle population has expanded significantly over time, contributing substantially to global milk production. Given their importance, it is crucial to examine their susceptibility to H5N1 infection and compare the findings to those observed in previous Holstein cows’ reports.

In our study, three Jersey cows were inoculated intranasally and intramammarily with a human H5N1 virus detected in Texas. Consistent with previous findings, milk production decreased rapidly in all three infected cows and remained low for at least seven days after inoculation. Although our observation period was short, studies of Holstein cows with longer observation periods have demonstrated that milk production did not return to normal levels, even after 23 days post-inoculation ^13^. It is important to note that the control cow (cow #1) exhibited a reduction in milk production upon transport to the BSL-3 laboratory. However, milk production levels returned to baseline within three days. This decline was likely caused by the stress of isolation and the unfamiliar ABSL3 environment.

Milk samples from the infected quarters exhibited an abnormal, yellowish colostrum-like appearance, consistent with field observations, and presented with high CMT scores throughout the study. It is important to note that we also detected elevated CMT scores in the non-infected rear right teat of cow #2, as well as the front left and rear right teats of cow #3 and cow #4. Despite the ABSL3 room being cleaned four times daily, mastitis remains a prevalent disease among dairy cows. Our findings confirm that H5N1 was not the cause of mastitis in these cases, as we did not identify vRNA in the non-infected samples.

All three Jersey cows exhibited high fever peaks on day 1 post-inoculation, with their temperatures returning to normal levels within 2 days post-inoculation. Subsequent fever spikes were observed in cow #2 between days 3 and 4 post-inoculation and in cows #3 and #4 between days 5 and 6. Similarly, Holstein calves and cows experienced fever peaks within two days of inoculation, returning to normal temperatures thereafter ^20^. The delayed fever peaks observed in our study may be linked to the higher CMT scores observed as the study progressed, suggesting a more severe form of bacterial mastitis.

Transponders inserted in the udders of the four cows allowed us to monitor quarter temperatures over time. While overall temperatures of infected quarters decreased following H5N1 infection, data consistency varied among the cows. However, this temperature reduction may be linked to the overall decrease in milk production from these quarters. This is further supported by the decrease in milk production observed in cow #1, which appears to correlate with a decrease in temperature across all quarters on day 1. Although we do not have data on milk production for each quarter, we frequently encountered difficulties collecting milk samples from infected quarters, even in small volumes. This suggests that H5N1 can effectively replicate within the mammary gland, leading to significant tissue damage, a decrease in milk production, and consequently, a reduction in the quarter’s temperature.

Importantly, H5N1 infection in all three cows was confirmed by high levels of vRNA in various samples collected. By using a human H5N1 virus, we demonstrated that cows could be infected with a human H5N1 strain. This phenomenon, known as reverse zoonosis, contributes to the increased genetic diversity of swine influenza viruses, posing significant challenges for vaccine development and control efforts ^21^. Surprisingly, we detected vRNA on the external surface of infected teats in all three cows. This finding suggests a possible mechanism for the human cases linked to the dairy cattle outbreak. The presence of vRNA, and potentially low levels of infectious virus, could have facilitated transmission during milking through direct contact with infected teats.

High levels of vRNA detected in internal swabs of infected teats in all three cows indicate efficient H5N1 replication within the mammary gland. This was further confirmed by the presence of high levels of vRNA in milk samples for at least 7 days post-infection.

Consistent with these findings, vRNA was also detected in the lactiferous ducts and glandular parenchyma of infected teats in all three cows. Notably, cow #4 exhibited the highest vRNA levels, suggesting a persistent presence of the H5N1 virus in the mammary tissues for at least 7 days post-infection. Our viral load data was corroborated by histopathological analysis of mammary gland tissues, which showed significant mastitis and ductitis in the infected quarters. Additionally, the pathological lesions were associated with elevated protein levels, as demonstrated by IHC, and increased viral RNA, as detected by ISH. Our findings align with the evidence of highly abundant α2,3 linked sialic acids receptors in the bovine mammary gland ^22^. Conversely, low levels of viral RNA were detected in the nasal swabs of infected cows throughout the study. Furthermore, tissue collection revealed limited amounts of vRNA in the nasal turbinates, trachea, and lungs, suggesting inefficient viral replication within the respiratory tract of Jersey cows. Consistent with previous findings, we did not detect vRNA in any of the other tissues collected in our study.

The unprecedented interspecies transmission of H5N1 clade 2.3.4.4b to various mammalian species, including dairy cattle with genotype B3.13, represents a significant milestone for the evolution of H5N1 viruses. As H5N1 viruses continue to infect new hosts, spread geographically, and reassort with other influenza subtypes, the risk of a pandemic steadily increases. Moreover, with the daily rise in infected dairy cattle herds, the likelihood of H5N1 becoming endemic in cattle increases correspondingly. Overall, our findings indicate that Jersey cows are susceptible to infection with a human H5N1 virus. The elevated vRNA levels detected in mammary gland tissues confirm the tissue tropism of H5N1 for the bovine mammary gland, and, to a lesser extent, the respiratory tract. All infected cows experienced fever peaks, significant reductions in milk production, and mastitis following infection. Our results suggest that Jersey cows serve as a valuable experimental model for investigating H5N1 disease pathogenesis and developing effective vaccines. Furthermore, the risk of reverse zoonosis could potentially increase the genetic diversity of H5N1 in cows, thereby raising the risk of a pandemic.

## Supplementary Material

Supplementary Table

Supplementary 1 and 2 are not available with this version.

This is a list of supplementary files associated with this preprint. Click to download.

• FigureS1.tiff

• FigureS2.tiff

• FigureS3.tiff

• FigureS4.tiff

• FigureS5.tiff

## Figures and Tables

**Table 1:** Tissues collection from all cows on days 5 (cow #2), 6 (cow #3), and 7 (cow #4) post-infection at the time of necropsy.

Major system	Tissues
Respiratory	BALF, diaphragm, **turbinate (ventral concha and ethmoid), trachea, larynx, lungs (accessory lobe, caudal left lobe, caudal part left cranial lobe, caudal part right cranial lobe, caudal right lobe, cranial part left cranial lobe, cranial part right cranial lobe, middle lobe)**
Digestive	Abomasum, omasum, reticulum, rumen, jejunum, ileum, descending colon, spiral colon, pancreas, **liver**, feces
Reproductive	Uterus, ovary, vagina, oviduct
Lymphatic	LN Ileocecal, LN Inguinal, LN Mandibular, LN Mesenteric, LN Parotid, LN Popliteal, **LN Retropharyngeal, LN Supramammary**, LN Tracheobronchial
Nervous	Brainstem, **cerebellum, cerebrum, hypothalamus, hypophysis**
Mammary	**Mammary front left (mucosa and body), mammary rear left (mucosa and body), mammary front right (mucosa and body), mammary rear right (mucosa and body)**
Muscles	Rump (gluteus medius, minimus, biceps femoris), tenderloin, Brisket (deep + superficial pectoral)
Other	Conjunctiva, heart, kidney, ocular fluid aqueous, ocular fluid vitreous, spleen
Tissues highlighted in bold were also collected for pathology analyses.	

## Data Availability

All data generated or analyzed during this study are included in this published paper and are available upon request.
